# Rice Farmers' Knowledge of the Risks of Pesticide Use in Bangladesh

**DOI:** 10.5696/2156-9614-8.20.181203

**Published:** 2018-12-06

**Authors:** Muhammad Matiar Rahaman, Khandakar Shariful Islam, Mahbuba Jahan

**Affiliations:** 1 Department of Agriculture, Pakutia College, Ghatail, Tangail, Bangladesh; 2 Department of Entomology, Faculty of Agriculture, Bangladesh Agricultural University, Mymensingh, Bangladesh

**Keywords:** rice farmers, pesticides, environment, ecosystem, Bangladesh

## Abstract

**Background.:**

Population growth has led to the need to increase global food production. Pesticides are an important tool used in efforts to control insect pests. About 20–30% of agricultural produce is lost annually due to insect pests, diseases, weeds and rodents. While pesticides are effective against pest populations, if used injudiciously, they may pose health hazards to humans, domestic animals, natural enemies of crop pests and other forms of life through unwanted contamination of food, feed, water bodies and the environment.

**Objectives.:**

The aim of the present study was to examine farmers' level of knowledge and awareness of environmental pollution due to unsafe use of pesticides for controlling rice pests and to explore ways of reducing their usage.

**Methods.:**

The research population consisted of 120 rice farmers: 40 farmers randomly selected from each of the three rice growing districts of Bangladesh; Mymensingh, Tangail and Jamalpur. Data was collected through group discussions, direct observations and personal interviews during March 2013 to May 2014.

**Results.:**

Farmers mainly sought advice on pesticide use from pesticide dealers or retailers and a very few farmers contacted government extension workers for this purpose. Most of the farmers had an understanding of natural enemies of rice pests and that the application of synthetic insecticides in the field can reduce their population. A few farmers followed integrated pest management (IPM) practices with little understanding of the adverse effects of insecticides on the environment and ecosystem. The majority of farmers understood the harmful effects of pesticides on health of human and animals, beneficial species, fish, insect resistance, soil and food. It was also found that education on pest management, information through television, more contact with extension personnel and farmers' awareness of IPM were critical factors for improving rice farmers' understanding of the ecological hazards caused by overuse of pesticides. Most of the farmers suggested that timely removal of weeds, appropriate timing for pesticide application, balanced doses of fertilizers, pest monitoring, correct dose of appropriate pesticides, pest tolerant varieties, increasing technical knowledge and skills and creating social awareness of environmental pollution among farmers were necessary to reduce the quantity of pesticides and minimize environmental hazards.

**Conclusions.:**

The present study identified a need to intensify farmers' awareness and knowledge of integrated pest management and environmental pollution through extension organizations which could help promote sustainable agricultural development and improve the currently endangered bio-diversity of Bangladesh.

**Participant Consent.:**

Obtained

**Ethical Approval.:**

This study was approved by the PhD Supervisory Committee assigned by the Department of Entomology of Bangladesh Agricultural University.

**Competing Interests.:**

The authors declare no competing financial interests

## Introduction

Demand for rice in Bangladesh is rapidly increasing along with an increase of population and rice demand in Bangladesh is currently the highest among developing countries.^1^ In order to increase rice production, farmers are using modern varieties of rice, along with intensive use of fertilizers, pesticides, water and other technologies which have changed the ecology and escalated pest proliferation.^2^ Insect pests are yield reducers and have increased many folds over recent decades. Pesticides used in the paddy fields globally account for nearly 15% of the total pesticides used for crop production.^3^

Pesticides are an important and reliable tool in an integrated pest management (IPM) program to curtail crop losses. In the past, their indiscriminate use has polluted the environment through harmful accumulation in food, feed, soil, water bodies, and air. A sensible approach to increase crop production with the use of pesticides calls for their judicious use as part of an established IPM program. Therefore, it is necessary to be aware of the economic costs of pests, selection of appropriate pesticides, dose and formulation, as well as the effects of pesticides on the environment. Studies have addressed many aspects of pesticide use, from purchase to various stages of their application and use. Safe and effective use of pesticide can help achieve the target of sustainable and environmentally friendly agricultural production.

Growth in global population means that farmers must produce food for an estimated 9.1 billion people expected to inhabit the earth by 2050. To feed this growing population, food production must be scaled up by 70% and this may be achieved through proper utilization of available plant genetic resources by developing high yielding varieties of crops, and improving crop production and protection technologies. Pesticides are an important tool used in efforts to control insect pests. About 20–30% of agricultural produce is lost annually due to insect pests, diseases, weeds and rodents. Therefore, judicious use of pesticides plays a major role in plant protection. Pesticides are highly effective, rapid in action, convenient to apply, and are powerful and economical tools in pest management.

Farmers habitually apply fertilizers and hazardous insecticides in high quantities without assessing the actual field requirements due to inadequate knowledge. This indiscriminate input can accelerate insecticide resistance, pest resurgence, and secondary pest outbreak, leading to environmental contamination, persistent residual toxicity and destruction of beneficial insects.^4–11^ In the absence of a natural enemy population, the pest population can then multiply and enhance the extent of yield loss.^12^,^13^ Despite these drawbacks, synthetic insecticides are still the primary method used to control rice insects. Against this back drop, a new generation of insecticide molecules are reported to be safer for human health.^14^,^15^ Cultural, mechanical and physical practices of pest control with low chemical input form one of the most effective approaches for reducing insect pests in rice under a modern IPM methodology.^16^ Integrated pest management also encourages the multiplication of a natural enemy population for effective pest suppression.^17^ While pesticides are effective against the pest population, if used injudiciously, they may pose serious health hazards to humans, domestic animals, natural enemies of crop pests and other forms of life through unwanted contamination of food, feed, water bodies and the environment.

**Abbreviations**
*IPM*: Integrated pest management

It is therefore important that the pest management practices of farmers be improved by examining current practices. The aim of the present paper is to examine the level of farmers' knowledge about environmental pollution resulting from pesticide usage and factors influencing this knowledge and to explore alternative ways of reducing pesticides in rice cultivation.

## Methods

In order to carry out this study, a survey was conducted in three ricegrowing districts in Bangladesh, Mymensingh, Tangail and Jamalpur, from the period of March 2013 to May 2014.

### Study location

Tangail is a district in the central region of Bangladesh and lies between 24° 01′ and 24° 47′ north latitude and 89° 44′ and 90° 18′ east longitude. Tangail has a tropical climate, with less rainfall in winter than in summer. The temperature in Tangail averages 25.5°C. About 1872 mm of precipitation falls annually. The population of the district is about 3.8 million, with an area of 3,414.28 km^2^. Agriculture is the main occupation of the Tangail district. About 49.53% of residents are involved with agricultural activities.^18^ Its main agricultural products are paddy, potato, jute, sugarcane, sesame, linseed, wheat, mustard seed and pulse. The main fruit products are mangos, jackfruit, bananas, litchis, and pineapples.

The district of Mymensingh has an area of 4363.48 km^2^, located in between 24°15′ and 25°12′ north latitude and in between 90°04′ and 90°49′ east longitude. The average temperature is 25.22°C with an average rainfall of 2.249 mm. The total population is 4,489,726 and the main source of income for residents is agriculture (64.14%).^19^

The district of Jamalpur lies between 24°34′ and 25°26′ north latitude and between 89°40′ and 90°12′ east longitude. The annual average temperature of this district varies from 33.3°C to 12°C. Annual average rainfall is 2174 mm. The total area of the district is 2115.16 km^2^ of which 18.16 km^2^ is under forest. Paddy, jute, sugarcane, mustard seed, peanut, wheat, sweet potato, tobacco, betel leaf, chilly, pulse vegetables, etc. are main fruits of this district.^20^

### Data collection

Data were collected through the use of a standard, fully structured and objective oriented questionnaire which was prepared as the base document for the interview to avoid bias. The questionnaire can be found in Supplemental Material. The questionnaire was refined with knowledge and ideas from previously conducted survey research.^2^, ^21^, ^22^ All subjects gave informed consent. This study was approved by the PhD supervisory committee assigned by the Department of Entomology of Bangladesh Agricultural University. The survey measured awareness of the effects of pesticides. Forty rice farmers were randomly and purposively selected for the interview from each of the three locations for a total of 120 respondents. Farmers involved in rice cultivation in both the Amon (rain fed) and Boro (irrigated) seasons were selected for the survey and detailed information was obtained concerning farmers' knowledge and attitudes on the usage of pesticides in rice fields and their effects on the environment and ecosystems. The theme of the questions centered on farmers' use of insecticides to control rice pests. The scope of such a survey need not to be limited to farmers' pest management knowledge, attitudes and practices, but could also cover issues such as decision making patterns, agronomic practices and socio-economic profile. Therefore, after administering the initial questionnaire, the interviewer also obtained some information regarding farmers' socioeconomic level. The questionnaire was translated into Bengali and adapted to reflect local situations. First, the interviewer gave farmers a brief introduction to the survey objectives and explained that the survey data would be used for research and solicited their cooperation. The questionnaires were administered separately at all study locations by asking respondents questions one by one in a sequential order. Interviews were recorded. The data sheets were then collected. In most cases, the data collector went to the farmers' rice fields where farmers were working. Data were then compiled and processed.

## Results

Demographic information and basic characteristics of the farms are outlined in Table 1.

**Table 1—:**
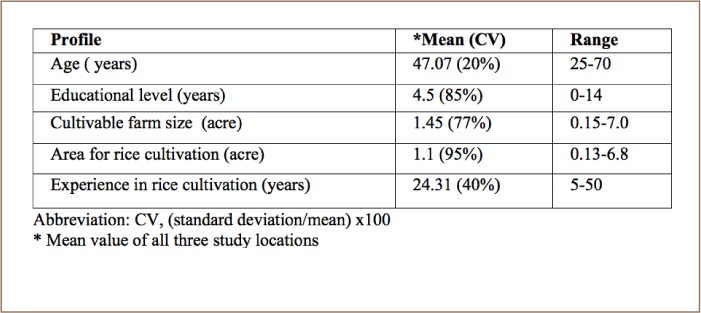
Demographic Characteristics of Farmers (n=20)

### Sources of advice for pesticide selection

Farmers' reported sources of advice on pesticide selection are presented in Table 2.

**Table 2:**
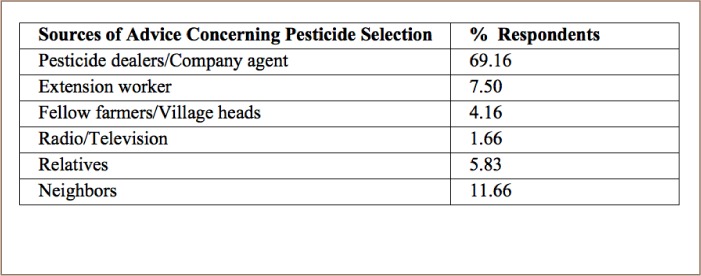
Sources of Advice on Pesticide Use for Farmers

### Farmers' knowledge of natural enemies of rice pests

Out of 120 farmers interviewed, an average of 33.98% farmers reported possessed good knowledge about natural enemies to rice pests, 50.33% reported limited knowledge and 15.66% reported no knowledge about natural enemies (*Table 3*). Most of the farmers (59.16%) reported a good understanding of the concept of a group of organisms that are natural enemies to insect pests in rice fields. Many farmers were able to name several natural enemies of rice pests. The most common natural enemies mentioned by farmers were spiders, dragonflies, birds, snakes, frogs, and different types of beetle and flies. Of the farmers who recognized the presence of natural enemies, 35% could describe their role. They had good knowledge of the role of natural enemies and knew that natural enemies feed on other harmful insects and/or their eggs, larvae, nymph or pupae. A total of 89.16% (34.16%+ 55%) of the farmers were aware of the adverse effects of insecticides on natural enemies. This was also evident from the answer to the question of pest resurgence. One fourth of farmers understood that killing of natural enemies could increase pest infestations. However, farmers' responses to further questions on the effect of insecticide application on pest populations were poor.

**Table 3:**
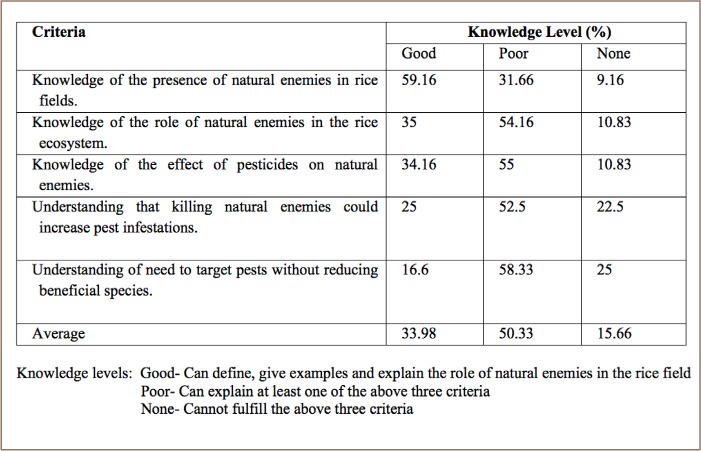
Farmers' Knowledge and Perceptions of Natural Enemies and the Toxic Effects of Pesticides

### Safety measures adopted for pesticide use

Safety measures used are summarized in Table 4. Almost all of the users reported reading the instructions printed on the pesticide containers and understood their danger and kept them out of reach of children. Among the farmers using pesticides in the field, almost all of them (90.83%) washed their hands with soap, a very few with soil, ash and water, 86.66% washed body after spraying, 66.66% disposed of empty pesticide containers, 68.33% protected domestic animals from going into sprayed rice fields and 32.5% took no safety measures before or after pesticide application.

**Table 4:**
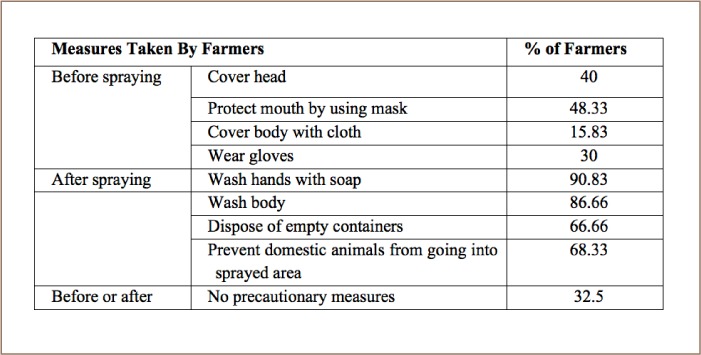
Safety Measures Adopted by Rice Growers for Pesticide Use

### Adverse effects of pesticides

Most farmers in the study area were conscious of the adverse effects of pesticides in the environment. Knowledge of adverse effects can be found in Table 5.

**Table 5:**
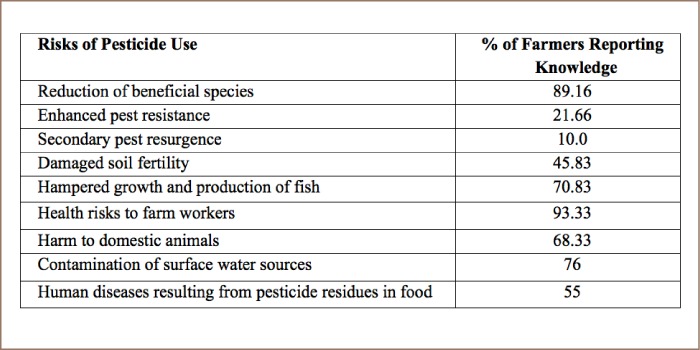
Farmers' Knowledge and Perception of Environmental Pollution Due to Pesticide Use (n=120)

### Factors influencing farmer's knowledge and perception of pesticide usage in Bangladesh

A summary of farmers' perception on the factors influencing knowledge of pesticide usage in Bangladesh can be found in Table 6.

**Table 6:**
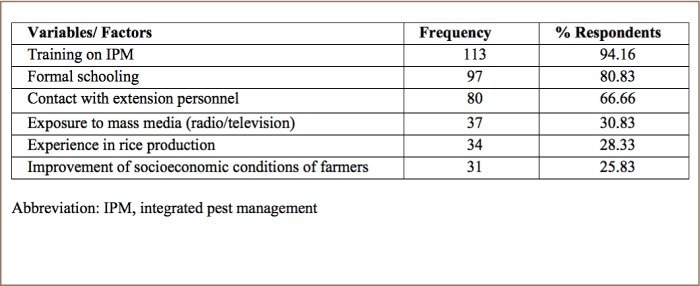
Factors Influencing Farmers' Knowledge and Perceptions of Pesticide Usage in Controlling Rice Pests

### Pesticide reduction measures

In the present study, respondents named ten ways of reducing pesticides (*Table 7*). More than half of the respondents (60.83%) placed an emphasis on timely removal of weeds, (58.63%) on appropriate timing for pesticide application, balanced doses of fertilizers (50.83%), pest monitoring, removal of egg masses and elimination of affected plants (45.83%), application of accurate doses of chemicals (36.66%), introduction of pest resistant varieties (34.16%), improving farmers' technical knowledge and skills (32.5%), raising social awareness of pesticide risks (30.83%), regulating the market of pesticides in mitigating the negative environmental impacts of agrochemical use (15.83%) and 12% of the farmers reported practicing integrated rice-fish agriculture that could help minimizing pesticide usage as well as mitigate environmental degradation. Providing information about the type, timing and volume of chemicals to farmers may improve their performance in this regard.

**Table 7:**
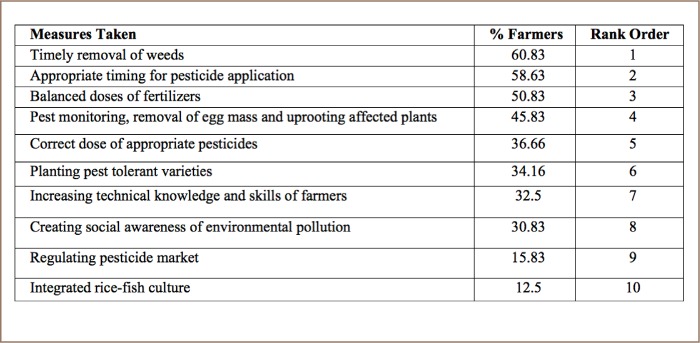
Pesticide Reduction Measures Reported by Farmers

## Discussion

Farmers' age, level of schooling, experience in rice cultivation and socioeconomic conditions were found to be major influential factors in their pest management decisions. Previous studies have attempted to analyze farmers' socio demographic profile and found that farmers' attitude and decisions on pest control strategies were guided by those factors.^2^,^23^ Previous studies found that farmers' education levels, training, experience, extension contacts and use of IPM methods were attributed to their level of awareness and knowledge about pesticides and the environment.^24–27^ The literate farmers in the survey area were eager to accept new technology. Educated farmers are quicker to accept recommended farm practices than their uneducated counterparts.^28^

### Sources of pesticide selection advice

Most of the farmers in the present study reported that they preferred to get advice on pesticide usage from pesticide dealers.^21^, ^29^–^31^ Reference groups or peer groups also significantly influenced farmers' decisions.^21^ Chemical company agents had the highest influence, followed by extension technicians, spouses, neighbors and village heads, respectively. Previous studies reported that about 61% of farmers received advice from pesticide dealers while selecting pesticides and their doses. In addition, 65% of farmers received advice from pesticide sales agents in selecting products and doses, 18% from neighbors, 8% from relatives, and the remaining 9% from extension workers.^31^

### Farmers' knowledge of natural enemies of rice pests

Farmers were aware of the effect of pesticides on natural enemies and they were very concerned about conservation of rice pest predators and parasitoids through use of pesticides that are less harmful to the natural enemies of rice pests. Botanicals and chemical insecticides have been found to be less harmful to the natural enemies of rice pests than chemical insecticides.^32^, ^33^

### Safety measures

The chemicals in many common insecticides can pose health risks to farm operators. Therefore, protective measures must be taken while spraying insecticides.^34^, ^35^ In the present study, most farmers reported taking some safety measures while handling chemicals to control rice insects. However, a smaller portion of farmers did not take any safety measures. These results are similar to those of prior studies.^36–38^ Lack of exposure to IPM and to new rice technologies were the main barriers to safety practices.^39^

### Harmful effects of pesticides

Pesticides can have harmful effects on ecosystems and the environment.^40^, ^41^ In the present study, a majority of farmers reported that extensive and indiscriminate use of chemicals caused health risks to farm operators.^34^, ^35^ Farmers were aware that pesticides applied in crop fields could reduce the population of beneficial species. Pesticides have been found to be largely responsible for the reduction in the number of natural pest predators such as earth worms, frogs, honey bees, spiders, flies, beetles, wasps, and other pollinating insects.^10^ Farmers were aware that pesticides could contaminate surface water, and it has been reported that 25% of pesticides used in agricultural areas in coastal districts might reach surface water systems as residue during the rainy season, cause hampered growth and production of fish, pose serious public health hazards, and lead to longterm pest resistance.^11^, ^42–47^ Farmers reported that insecticides can enhance secondary pest resurgence. This understanding is in agreement with previous studies that have reported that useful insects and animals are commonly killed as a consequence of pesticide use.^7^, ^48^ Livestock and poultry have also been poisoned or killed eating pesticide-affected grasses, straw or grain. Declining soil fertility reported by the farmers in the present study might be due to a gradual decrease in nutrient replenishment of soils due to destruction of useful microbes.

### Factors influencing farmer's knowledge and perceptions of pesticide usage in Bangladesh

The factors identified as potential factors enhancing farmers' knowledge levels about pest management strategies in Bangladesh in the present study are in agreement with previous studies that found that farmers' education level, farming experience, training on crop protection, extension contacts and use of IPM methods attributed to the level of awareness and knowledge about pesticides and environment.^24–27^ Norvell and Hammig reported a positive association between farmers with IPM training and knowledge of farm ecology.^49^ As shown in Table 2, data indicate that growers are not motivated to receive information from relevant sources such as the extension personnel of the Department of Agricultural Extension (Bangladesh). Only 7.5% of farmers reported that they got advice and information about pesticide use from extension workers, whereas 69.16% sought advice from pesticide dealers or retail sellers. Pesticide dealers and retailers play a vital role in the use of pesticides in Bangladesh. However, in the present study, 66.66% of farmers reported that contact with extension personnel would help improve their knowledge of proper pesticide usage (*Table 6*). This result (66.66%) is more or less contradictory with the findings presented in Table 2. Only 7.5% farmers contact extension worker as a source of pesticide usage advice while 69.16% of farmers take advice from pesticide dealers or company agents. Pesticide dealers do not have the expertise to provide guidelines to farmers for controlling insect pests effectively by using pesticides. Retailers are motivated to make a profit by selling specific pesticide products rather than being concerned about pesticide efficacy.

### Alternative methods of reducing pesticides

Many studies have examined alternative methods of pesticide application. In the present study, farmers reported ten ways of reducing pesticides. Farmers stressed the importance of using balanced doses of fertilizers and this is in agreement with previous studies that have suggested applying organic materials mixed with nitrogen fertilizer to minimize pest prevalence in the rice field.^50^, ^51^ Pest monitoring and removal of egg mass were also named as alternative ways of reducing pesticides by the farmers. This concept is supported by previous studies which have reported that around 40% of yield loss due to stem borers in rice could be avoided by either removal of egg mass or application of ovicides.^52^ Development and adoption of pest resistant varieties can also reduce farmers' dependence on harmful agrochemicals.^53^ The need to improve farmers' knowledge and practical skills in optimizing the use of pesticides on rice farms through training was also reported by farmers. This is in agreement with earlier findings on this topic.^24^, ^54^ In addition, the importance of the creation of mass awareness of the adverse effects of agro-chemical products and motivation to increase farmers' adoption of IPM technologies was reported by farmers. Previous studies have supported this assertion.^55^ The utility of integrated rice-fish culture was reported by farmers and numerous studies have reported that integrated rice-fish farming benefits the conservation of rice pest predators, requires smaller amounts of pesticide and fertilizers, promotes preserving a population of natural enemies for biological control of rice pests and ultimately provides a sustainable, economic and ecologically friendly form of pest control.^44^, ^45^ Lastly, previous studies have recommended that effective policy guidelines, pesticide regulation and its effective implementation, motivation of farmers and extension staff, effective environmental technology transfer, promotion of participatory oriented extension programs, awareness building through mass media, expansion of IPM practices, situation analysis and integration of local knowledge could prevent future deterioration of the agro-ecosystem and sustain overall development. 26, 56

## Conclusions

It is crucial that farmers gain increased awareness and knowledge of ecological hazards and IPM. Intensive training and practical demonstration under the supervision of extension personnel would help ensure successful reduction of pesticide use in rice cultivation. The results of the present study will assist in improving the action plan of development agencies by disseminating knowledge and technology among farmers to enhance rice production without depleting environmental quality. Further in-depth studies are needed to assess the environmental impacts of rice cultivation and to examine the relationship between IPM awareness and knowledge and their effects on farmers' adoption of environmentally friendly pest management technologies.
