# Supplementary material for: Rice Farmers' Knowledge of the Risks of Pesticide Use in Bangladesh
Source: J Health Pollut. 2018 Dec 6;8(20):181203. doi: 10.5696/2156-9614-8.20.181203 (PMC6285676; doi:10.5696/2156-9614-8.20.181203)
Supplement: Supplementary file 1 [file hapn-8-20-181203_s01.docx]

**Structured oriented questionnaire**

**Questionnaire for the study on farmers’ knowledge and perception of pesticide usage
in Bangladesh in controlling rice stem borer.**

Department of Entomology
Bangladesh Agricultural University, Mymensingh- 2202.

1. **Questionnaire for field survey:**

Date of interview:…………………………Farmer’s number:……………………………

Farmer’s name:………………………………………………

District…………………… Upazilla……………………

Union…………………… Village……………………

**Farmer’s information**

1. Gender: Male…… Female……

2. Age: …… 3. Level of education…………………………

4. Agricultural farming experience……………………years

5. Rice farming experience…………………………years

6. Total cultivable land…………………………

7. Rice cultivated land (acre) …………………………

8. Variety cultivated: Amon…………………………Boro…………………………

**B. Information about pest management**

9. What pests of rice do you have in the field?

a) ………………………… b) ………………………… c) …………………………

d) ………………………… e) ………………………… f) …………………………

10. Do you know about rice stem borer? ……Yes ……No

11. If yes, how can you identify the pest? ……………………………………

12. When do you think the damage is worst? ……………………………………

13. What percentage of your total rice production was damaged by stem borer?

During: last Boro season (2012) ……%, Amon season (2012) ……%

Which one is important for controlling stem borer?

1. Need to control
2. Difficult to control
3. Easy to control.

14. How did you control this pest? ()

…… Only insecticides, ……IPM (hand picking, botanicals) …… or ……Other (s)

If other method, please specify: ……………………………………………………

15. Mention the insecticides which you apply in the rice field

| Name of insecticides | Did you use before | If yes, how many years | Pesticide group  (not for the farmers to answer) |
| --- | --- | --- | --- |
| 1 |  |  |  |
| 2 |  |  |  |
| 3 |  |  |  |
| 4 |  |  |  |
| 5 |  |  |  |
| 6 |  |  |  |
| 7 |  |  |  |
| 8 |  |  |  |
| 9 |  |  |  |
| 10 |  |  |  |

16. From where you get pest control advice?:

1. Pesticide dealers/ Company agent
2. Extension worker
3. Fellow farmers/village heads
4. Radio/ TV
5. Relatives
6. Neighbors

17. How many times you applied insecticides?

(a) Once

(b) Twice

(c)Thrice

18. How many insecticides you applied?

(a) Insecticide one

(b) Insecticide more than one.

19. What are the forms of insecticides?

(a) Single

(b) Mixture

20. What doses do you follow when apply?

(a) Recommended dose

(b) Over dose

21. Do you bring any change in using insecticide?

(a) Same insecticide used

(b) Alternative insecticide used.

21. How many times you apply urea fertilizer?

(a) Once

(b) Twice

(c) Thrice

22. Do you think urea fertilizer enhance stem borer in rice field?: ---------yes,----------no.

If yes, why?

23. When did you take action to apply insecticides---

(i) At cropping stage?

(a) Tillering stage or earlier

(b) Vegetative stage

(c) Booting stage

(d) Heading stage or later

(ii) At insect growth stage?

1. Egg (b) Larva (c) pupa (d) adult.

(ii) At infestation stage?

1. Dead heart (b) White head (c) Before symptom seen (d) Routine use.

23. What time in the day is more effective you think for spray?

(a) Morning (b) Noon (c) Afternoon.

24. Do you take any precaution measures before or after spraying insecticides?

1. Covering head
2. Protecting mouth by using musk
3. Covering body with cloth
4. Using hand gloves
5. Washing hand with soap
6. Washing body
7. Disposing empty containers
8. Protecting domestic animals
9. No precaution measure

25. What percentage of stem borer is killed by spraying insecticides?

1. < 50 % (b) 50-75 % (c) 75 %>

26. What percentage of infestation (DH and WH) was reduced by the insecticides?

(a)< 25 % (b) 50-75 % (c) 75 %>

27.What is your overall assessment on the effectiveness of the insecticides

1. Good effective (b) moderately effective (c) less effective (d) not effective

28. Rice of which season are more affected?

(a) Aus (b) Amon (c) Boro

29. which varieties are more susceptible?

Amon:------------Boro------------------

29. Do you have any insect not harmful to your crop?: -----------yes------------no.

(If yes), (a) Can you tell their name?

(b) Do you know their role in rice field?

(c) What was the plight of those insects after applying insecticides?:---------they were destroyed---------disappeared--------do not know------other

(d)Application of insecticides, even also natural enemies of pest could be destroyed- are you

-----agree------disagree-----------no comments.

(If agree), killing of natural enemies increase pest infestation- are you

--------agree---------disagree-------no comments.

Do you have any idea on killing stem borer without reducing those beneficial insects?

-------yes,------------no

If yes, please specify:------------------------.

30. What is your opinion about the bad effects of using insecticides? (Tick Marks)

Reduction of beneficial species/Enhance pest resistance and/Secondary pest resurgence/ Damaging soil fertility/ Hampering growth and production of fish/ Causing health risk to farm workers/Harmful to domestic animals/Contaminating surface water sources/Adding toxic elements in underground water/Causing human diseases due to pesticide residues in food.

31. What is your opinion about the bad effects of using insectides? (Tick Marks)

Balanced doses of fertilizers/Pest tolerant varieties/Regulating insecticides market/Correct dose of appropriate insecticides/ Pest monitoring, removal of egg mass and uprooting affected plants/Increasing technical knowledge and skills of farmers/Timely removal of weeds/Appropriate timing for insecticide application/Integrated rice-fish culture/Creating social awareness of environmental pollution.

32. What is your opinion about factors influencing farmer’s knowledge and perception about insecticide use? (Tick Marks)

Formal schooling/Training on IPM/Contact with extension personnel/Exposer to mass media (Radio/TV)/Experience in rice production/Socioeconomic condition of the farmers.

33. Estimation of cost-benefit ratio (one farmer from each of the 3 locations to be interviewed in both Amon and Boro season)

a) How many times you have sprayed

b) How much you have spent per acre or bhiga for each of the spray

c) Number of WH in 1 sq.m area will be counted in the sprayed and unsprayed field and other yield data in the unit area necessary to calculate crop benefit ratio in the farmer’s field.

Name of the Researcher: Name of the Supervisor:

(Muhammad Matiar Rahaman) (Dr. Khandakar Shariful Islam)

PhD Fellow Professor
